# A Multidomain Prediction Model Integrating Myocardial Injury, Ventricular Function, and Inflammation for Short-Term Risk Stratification in Patients with NSTEMI

**DOI:** 10.3390/clinpract16080143

**Published:** 2026-08-04

**Authors:** Emir Bećirović, Minela Bećirović, Amir Bećirović, Amir Tursunović, Ajla Bajrić, Amil Softić, Adna Mujkić, Elma Mujaković, Admir Abdić, Lamija Ferhatbegović

**Affiliations:** 1 Department of Internal Medicine, University Clinical Center Tuzla, 75000 Tuzla, Bosnia and Herzegovina; minela03@live.com (M.B.); amirbecirovictz@gmail.com (A.B.); 2 Department of General and Abdominal Surgery, University Clinical Center Tuzla, 75000 Tuzla, Bosnia and Herzegovina; tursunovicamirmd@gmail.com; 3 Primary Health Care Center Tuzla, 75000 Tuzla, Bosnia and Herzegovina; ajlabajric458@gmail.com; 4 Department of Plastic and Reconstructive Surgery, University Clinical Center Tuzla, 75000 Tuzla, Bosnia and Herzegovina; dr.amil.softic@gmail.com; 5 Faculty of Medicine, University of Tuzla, 75000 Tuzla, Bosnia and Herzegovina; adna.mujkic@untz.ba (A.M.); elma.mujakovic@untz.ba (E.M.); 6 Department of Abdominal Surgery, Cantonal Hospital Bihać, 77000 Bihać, Bosnia and Herzegovina; ab.admir@gmail.com; 7 Department of Internal Medicine, Cantonal Hospital Zenica, 72000 Zenica, Bosnia and Herzegovina; lamija.pojskic@gmail.com

**Keywords:** non-ST-segment elevation myocardial infarction, high-sensitivity cardiac troponin I, C-reactive protein, left ventricular ejection fraction, major adverse cardiovascular events, risk stratification, prediction model

## Abstract

**Background/Objectives**: Early risk stratification remains challenging in patients with non-ST-segment elevation myocardial infarction (NSTEMI). The present study evaluated the prognostic value of 24 h high-sensitivity cardiac troponin I (hs-Troponin I) and assessed whether combining biomarkers and echocardiographic parameters improves short-term risk prediction. **Methods**: This prospective observational cohort study included 170 consecutive adult patients with confirmed NSTEMI who were admitted to a Medical Intensive Care Unit and prospectively enrolled between February 2022 and January 2023. Clinical, routine biochemical, inflammatory, hematological, lipid, and echocardiographic data were collected during index hospitalization. High-sensitivity cardiac troponin I was measured at admission and again 24 h after hospitalization, with the 24 h value used as the principal marker of myocardial injury in the prediction analyses. The primary endpoint was major adverse cardiovascular events (MACEs), defined as cardiovascular death, recurrent myocardial infarction, ischemic stroke, urgent coronary revascularization, or hospitalization for worsening heart failure, within 3 months. Multivariable logistic regression, Cox regression, sequential prediction modeling, and internal bootstrap validation were performed. **Results**: MACEs occurred in 88 patients (51.8%). Twenty-four-hour hs-Troponin I, but not admission hs-Troponin I, was independently associated with MACEs (OR 1.57, 95% CI 1.09–2.26; *p* = 0.015) and a shorter time to the first MACE event (HR 1.38, 95% CI 1.07–1.78; *p* = 0.012). Lower left ventricular ejection fraction (LVEF) was also independently associated with adverse outcomes. The addition of 24 h hs-Troponin I, LVEF, and C-reactive protein improved discrimination from an AUC of 0.665 to 0.759 (optimism-corrected AUC, 0.717), with corresponding improvements in reclassification. A simplified multimarker score was independently associated with event-free survival (HR 2.36, 95% CI 1.53–3.64; *p* < 0.001). **Conclusions**: In patients admitted to a medical intensive care unit with NSTEMI, the integration of 24 h hs-Troponin I, LVEF, and C-reactive protein improved short-term risk prediction beyond that of clinical variables alone. A practical multimarker model based on routinely available parameters identified patients at increased risk of adverse cardiovascular outcomes during early follow-up.

## 1. Introduction

Non-ST-segment elevation myocardial infarction (NSTEMI) is the most common presentation of acute coronary syndrome and remains associated with a substantial risk of recurrent cardiovascular events despite contemporary treatment strategies [1]. Patients admitted to medical intensive care units with NSTEMI represent a particularly vulnerable population because they require continuous monitoring and timely therapeutic interventions, while clinical deterioration may occur despite initially stable presentation [2]. Early identification of patients at increased risk of adverse outcomes is therefore a key component of management, influencing monitoring strategies, clinical decision-making, and post-discharge follow-up [3]. However, outcomes vary considerably among patients with apparently similar clinical characteristics, highlighting the need for improved risk stratification [4].

Cardiac troponins are central to the diagnosis of myocardial infarction and are widely used for prognostic assessment [5]. Higher troponin concentrations generally reflect greater myocardial injury and have been associated with adverse cardiovascular outcomes [6]. Nevertheless, prognosis after NSTEMI is influenced by several interrelated pathophysiological mechanisms. Inflammatory activation contributes to plaque instability, recurrent ischemic events, and adverse ventricular remodeling, while left ventricular systolic dysfunction reflects the functional consequences of myocardial injury and remains one of the strongest predictors of adverse cardiovascular outcomes [7]. In critically monitored patients, serial biomarker assessment may provide a more accurate estimate of myocardial injury burden than measurements obtained at presentation alone, which are influenced by variability in symptom onset and timing of hospital admission [8].

Although biomarkers reflecting myocardial injury, inflammation, and ventricular dysfunction have demonstrated prognostic value, their performance is often modest when evaluated individually [9]. Consequently, increasing attention has been directed toward multimarker approaches that integrate complementary information from different biological domains in order to improve risk prediction and identify patients at greatest risk of early clinical deterioration [10]. Despite advances in risk stratification, data regarding practical multimarker strategies based on routinely available clinical variables remain limited among patients requiring intensive care unit admission [11].

The aim of the present study was to evaluate the prognostic significance of 24 h high-sensitivity cardiac troponin I (hs-Troponin I) concentrations and to determine whether the integration of routinely available biomarkers and echocardiographic parameters reflecting myocardial injury, ventricular function, inflammation, and metabolic status improves the prediction of major adverse cardiovascular events (MACEs) during short-term follow-up after NSTEMI, as well as to develop a simplified multidomain risk score suitable for routine clinical practice. In addition, we sought to develop a practical, multimarker prediction model based on routinely available clinical, laboratory, and echocardiographic variables to facilitate early identification of high-risk patients admitted to a medical intensive care unit.

## 2. Materials and Methods

### 2.1. Study Design and Population

This study represents a secondary analysis of data derived from a previously conducted prospective observational cohort study performed in the Medical Intensive Care Unit of the Clinic for Internal Medicine at the University Clinical Center Tuzla, Bosnia and Herzegovina, a tertiary referral center providing advanced care for critically ill patients with acute cardiovascular and other internal medicine conditions. The original prospective cohort was conducted between February 2022 and January 2023. The Medical Intensive Care Unit is a multidisciplinary tertiary-care unit providing continuous monitoring and advanced management for patients with acute cardiovascular, respiratory, renal, endocrine, and other life-threatening internal medicine disorders.

Consecutive patients aged ≥18 years with confirmed NSTEMI were prospectively enrolled between February 2022 and January 2023. Patients younger than 18 years, those without a confirmed diagnosis of NSTEMI, and those not admitted to the Medical Intensive Care Unit were not considered eligible for study enrollment. Exclusion criteria included active malignancy, autoimmune or systemic inflammatory disease, acute infection requiring systemic antimicrobial therapy, advanced chronic liver disease, end-stage renal disease requiring dialysis, major surgery or trauma within four weeks before admission, and incomplete clinical, laboratory, echocardiographic, or follow-up data.

NSTEMI was diagnosed according to contemporary international criteria based on symptoms or objective evidence of myocardial ischemia, elevated cardiac troponin concentrations above the 99th percentile upper reference limit, and the absence of persistent ST-segment elevation on electrocardiography (ECG). The study population comprised consecutive adult patients presenting with NSTEMI who required admission to the Medical Intensive Care Unit for specialized management. A total of 170 patients fulfilled the study criteria and were included in the final analysis.

### 2.2. Clinical, Laboratory, and Echocardiographic Assessment

Demographic characteristics, cardiovascular risk factors, medical history, and treatment data were obtained from hospital records and standardized clinical documentation. Arterial hypertension (HTN) was defined as a previous diagnosis of hypertension or ongoing antihypertensive therapy, while diabetes mellitus (DM) was defined as a previous diagnosis or active glucose-lowering treatment. Smoking status was categorized as active smoking or non-smoking at admission. Body mass index (BMI) was calculated as weight in kilograms divided by height in meters squared.

Venous blood samples were obtained at admission and at 24 h after hospitalization in accordance with institutional protocols. Hs-Troponin I concentrations were measured using the ARCHITECT i2000SR immunoassay analyzer (Abbott Diagnostics, Abbott Park, IL, USA). Routine biochemical analyses were performed using the AU5800 Clinical Chemistry Analyzer (Beckman Coulter, Brea, CA, USA). Routine laboratory analyses included glucose, creatinine, C-reactive protein (CRP), ferritin, lipid parameters, and complete blood count. The 24 h hs-Troponin I concentration was selected as the principal marker of myocardial injury burden. The 24 h measurement was selected a priori because it was considered more representative of cumulative myocardial injury than admission concentrations, which may be influenced by variability in symptom onset and presentation timing. Inflammatory status was assessed using CRP and ferritin concentrations measured 24 h after admission.

The triglyceride–glucose (TyG) index, triglyceride-to-high-density lipoprotein cholesterol ratio (TG/HDL-C), and atherogenic index of plasma (AIP) were calculated using standard formulas and evaluated as markers of metabolic dysfunction.

Transthoracic echocardiography (TTE) was performed immediately after hospital admission as part of the initial evaluation, typically within the first 30 min of presentation, using a Vivid T8 ultrasound system (GE HealthCare, Chicago, IL, USA). LVEF was assessed using the biplane Simpson method in accordance with contemporary echocardiographic recommendations. All echocardiographic examinations were performed by a single experienced operator; therefore, interobserver variability was not assessed. Additional measurements included left atrial diameter, interventricular septal thickness, and inferior vena cava diameter. All patients received guideline-directed treatment for NSTEMI in accordance with the contemporary European Society of Cardiology (ESC) guidelines for acute coronary syndromes without persistent ST-segment elevation, in addition to institutional protocols.

### 2.3. Outcome Definition and Follow-Up

Patients were followed for three months after index hospitalization. Follow-up information was obtained from hospital records, outpatient evaluations, and telephone contact when necessary.

The primary outcome was MACEs, defined as a composite of cardiovascular death, recurrent myocardial infarction, ischemic stroke, urgent coronary revascularization, or hospitalization for worsening heart failure. For time-to-event analyses, the interval between hospital admission and the first occurrence of any component of the composite endpoint was recorded. For patients experiencing more than one event during follow-up, only the first event was considered in time-to-event analyses.

### 2.4. Development of Prediction Models

To evaluate the incremental prognostic contribution of different pathophysiological domains, a series of sequential prediction models was constructed. The baseline clinical model included age, sex, HTN, DM, and active smoking status. Candidate variables were selected a priori based on clinical relevance and previously established associations with cardiovascular outcomes. Subsequent models incorporated 24 h hs-Troponin I, LVEF, and CRP sequentially. The variables included in the simplified four-component risk score were selected because they represented complementary pathophysiological domains (clinical risk, myocardial injury, ventricular dysfunction, and inflammation), demonstrated the strongest overall incremental prognostic contribution during sequential model development, and are routinely available in everyday clinical practice.

Model performance was evaluated using discrimination and reclassification metrics. In addition, a simplified multimarker risk score was developed using variables with the strongest overall prognostic contribution. Cut-off values for each component were determined by receiver operating characteristic analysis using the Youden index. One point was assigned to each variable exceeding its corresponding ROC-derived threshold, and the numerical cut-off values are presented in the Section 3. Patients were subsequently categorized into low-, intermediate-, and high-risk groups.

### 2.5. Statistical Analysis

Statistical analyses were performed using IBM SPSS Statistics software, version 26.0 (IBM Corp., Armonk, NY, USA). The distribution of continuous variables was assessed before statistical analyses. Variables with non-normal distributions are presented as medians with interquartile ranges (IQRs), whereas categorical variables are presented as counts and percentages. Continuous variables were compared using the Mann–Whitney U test and categorical variables using the chi-square test or Fisher’s exact test, as appropriate.

Receiver operating characteristic (ROC) analysis was used to evaluate the discriminative performance of individual biomarkers and prediction models, with optimal cut-off values determined using the Youden index.

Candidate variables for multivariable analyses were selected a priori based on their established clinical relevance, biological plausibility, and previously reported associations with adverse cardiovascular outcomes in patients with NSTEMI. Independent predictors of MACEs were identified using multivariable logistic regression analysis and reported as odds ratios (ORs) with 95% confidence intervals (CIs). Time-to-event analyses were performed using Kaplan–Meier curves, log-rank testing, and Cox proportional hazards regression, with results reported as hazard ratios (HRs) and corresponding 95% confidence intervals. Variables with markedly skewed distributions were logarithmically transformed before regression analyses when appropriate.

Incremental model performance was assessed using changes in the area under the receiver operating characteristic curve (AUC), integrated discrimination improvement (IDI), continuous net reclassification improvement (NRI), bootstrap internal validation, and Brier score. Internal validation was performed using bootstrap resampling with 1000 iterations. Bootstrap resampling was used to assess model stability and reduce the risk of overfitting. Decision curve analysis was performed to evaluate the potential clinical utility of the prediction models. All statistical tests were two-sided, and exact *p*-values are reported where applicable. Multicollinearity among variables included in the final multivariable model was assessed using variance inflation factors (VIF).

### 2.6. Ethical Considerations

The study protocol was approved by the Ethics Committee of the University Clinical Center Tuzla (Approval No. 02-09/2-97/21) and was conducted in accordance with the principles of the Declaration of Helsinki. The present study represents a secondary analysis of data collected within the approved research project. Therefore, additional patient consent was not required because the study used routinely collected clinical data and involved no additional interventions or procedures.

## 3. Results

### 3.1. Study Population and Baseline Characteristics

A total of 170 patients with NSTEMI were included in the final analysis. Complete clinical, laboratory, echocardiographic, and follow-up data were available for all participants. During the 3-month follow-up period, MACEs occurred in 88 patients (51.8%), whereas 82 patients (48.2%) remained event-free.

Patients who developed MACEs had a higher prevalence of arterial hypertension, lower left ventricular ejection fraction, and higher 24 h hs-Troponin I and CRP concentrations, whereas active smoking was less common in this group (Table 1).

### 3.2. Components of Major Adverse Cardiovascular Events

Among patients who experienced MACEs, hospitalization for worsening heart failure and cardiovascular death were the predominant adverse events, accounting for more than half of all events (Table 2).

### 3.3. Biomarker Performance and Independent Predictors of MACEs

Admission hs-Troponin I concentrations did not differ significantly between groups. In contrast, patients who developed MACEs had higher 24 h hs-Troponin I and CRP concentrations and lower LVEF, whereas ferritin and metabolic indices were not associated with outcome (Table 1).

Among individual biomarkers, 24 h hs-Troponin I demonstrated the highest, but overall modest, discriminative performance (AUC 0.618, 95% CI 0.531–0.702), followed by CRP (AUC 0.602) and LVEF (AUC 0.595). Ferritin, TyG index, TG/HDL-C ratio, and AIP demonstrated limited discriminative performance (Table 3).

Multivariable analyses identified 24 h hs-Troponin I as the most consistent predictor across logistic and Cox regression models. Higher 24 h hs-Troponin I concentrations were independently associated with both MACE occurrence and earlier event development. HTN was associated with increased risk, whereas age and active smoking demonstrated inverse associations. LVEF remained independently associated with MACEs in logistic regression but not in time-to-event analyses. CRP showed borderline significance in logistic regression and was not independently associated with time-to-event outcomes (Table 4).

### 3.4. Development and Validation of the Multidomain Prediction Model

**Figure 1:**
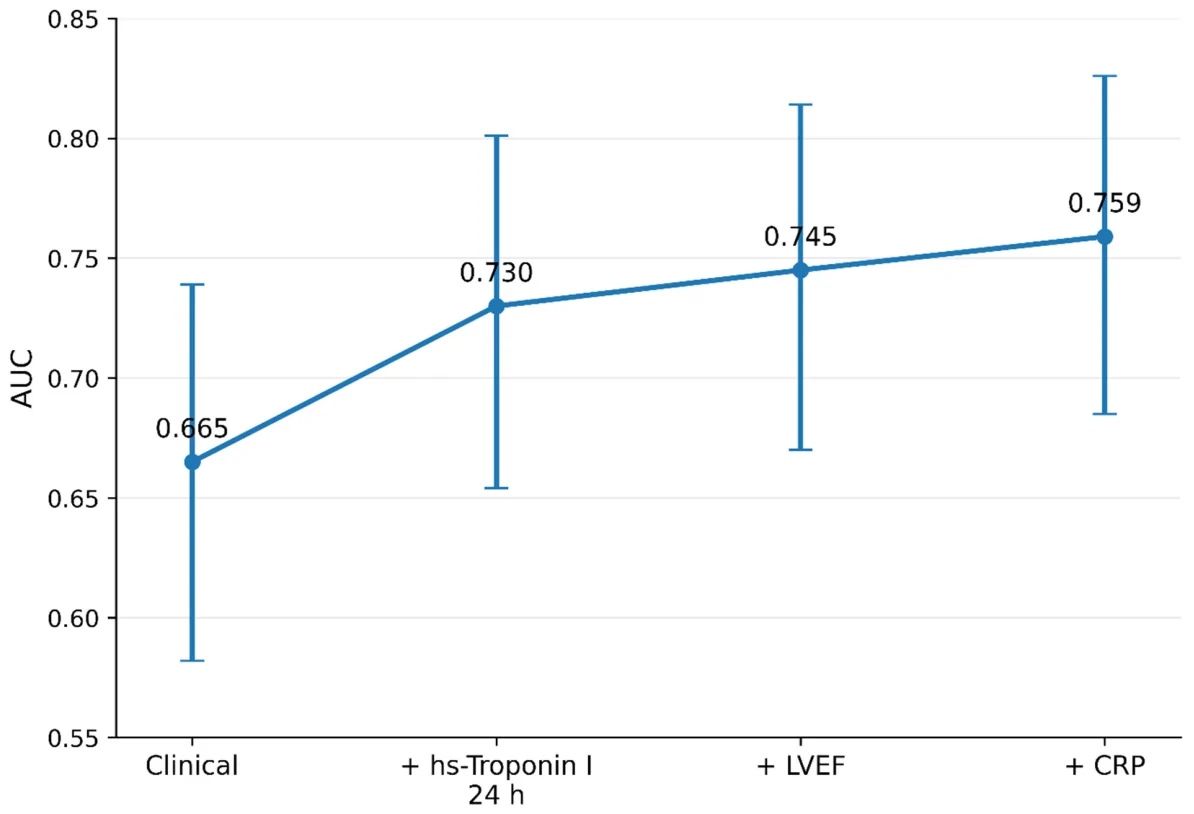
Receiver operating characteristic curves for sequential prediction models for 3-month major adverse cardiovascular events. Error bars represent 95% confidence intervals for the area under the curve (AUC).

The baseline clinical model demonstrated modest discrimination (AUC 0.665). The baseline clinical model included age, sex, arterial hypertension, diabetes mellitus, and active smoking. The final multidomain prediction model additionally incorporated 24 h hs-Troponin I, LVEF, and CRP (Table 5). The addition of 24 h hs-Troponin I increased the AUC to 0.730, the incorporation of LVEF increased it to 0.745, and the addition of CRP further improved discrimination to 0.759. At the optimal predicted probability cutoff of 0.527, the final multidomain model demonstrated a sensitivity of 72.7% and a specificity of 78.0%. The extended model, including ferritin and TyG index, achieved an AUC of 0.762 (Table 6, Figure 1). No evidence of multicollinearity was observed among the variables included in the final multidomain prediction model, with all variance inflation factors being below 1.4.

Bootstrap validation yielded an optimism-corrected AUC of 0.717 for the final multidomain model. The final multidomain model improved apparent AUC from 0.665 to 0.759 and optimism-corrected AUC from 0.623 to 0.717 compared with the clinical model. Predictive accuracy also improved, as reflected by a lower Brier score (0.201 vs. 0.229). The final model achieved an IDI of 0.106 and a continuous NRI of 0.586 (Table 7).

**Figure 2:**
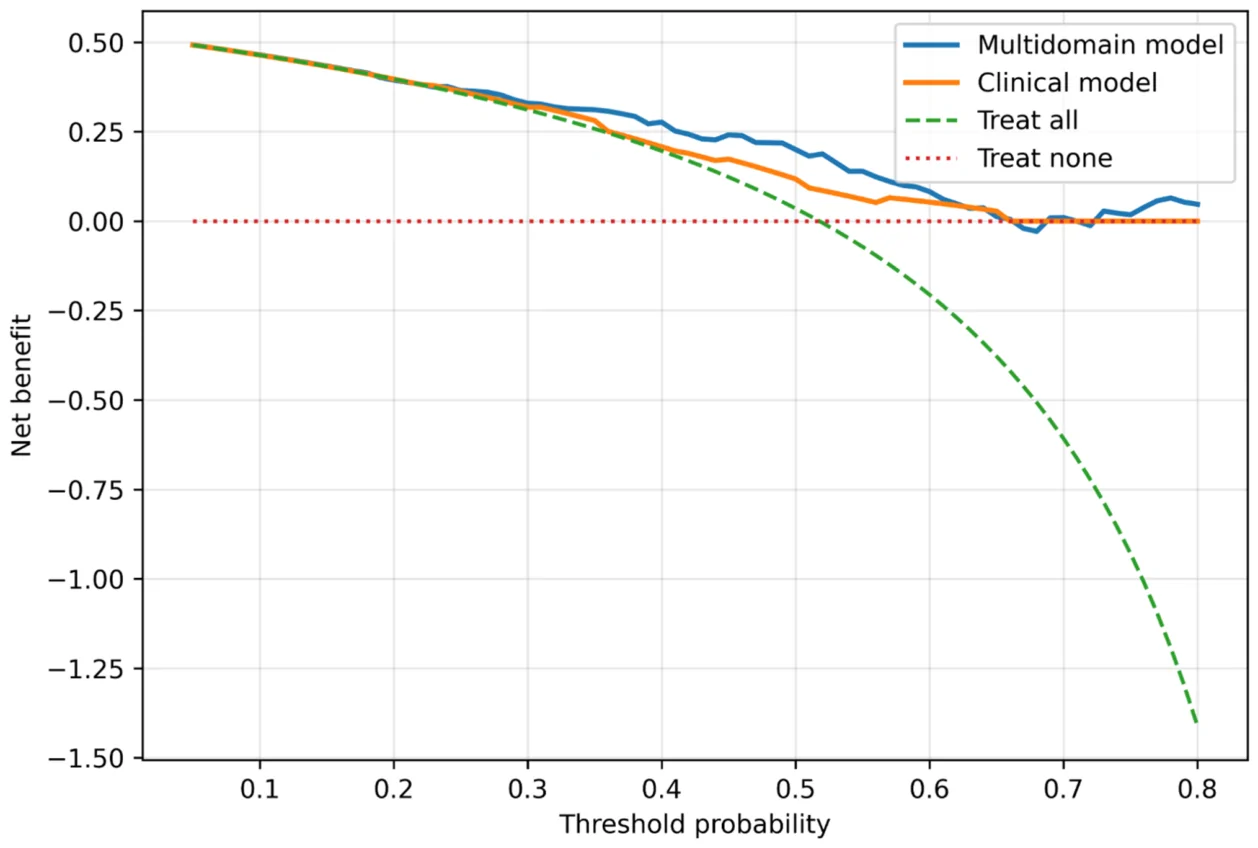
Decision curve analysis comparing the clinical and multidomain prediction models for predicting 3-month major adverse cardiovascular events. The multidomain model provided greater net benefit than the clinical model across a broad range of clinically relevant risk thresholds.

Decision curve analysis demonstrated greater net benefit for the multidomain model than for the clinical model (Figure 2).

### 3.5. Time-to-Event Analysis and Multimarker Risk Score

**Figure 3:**
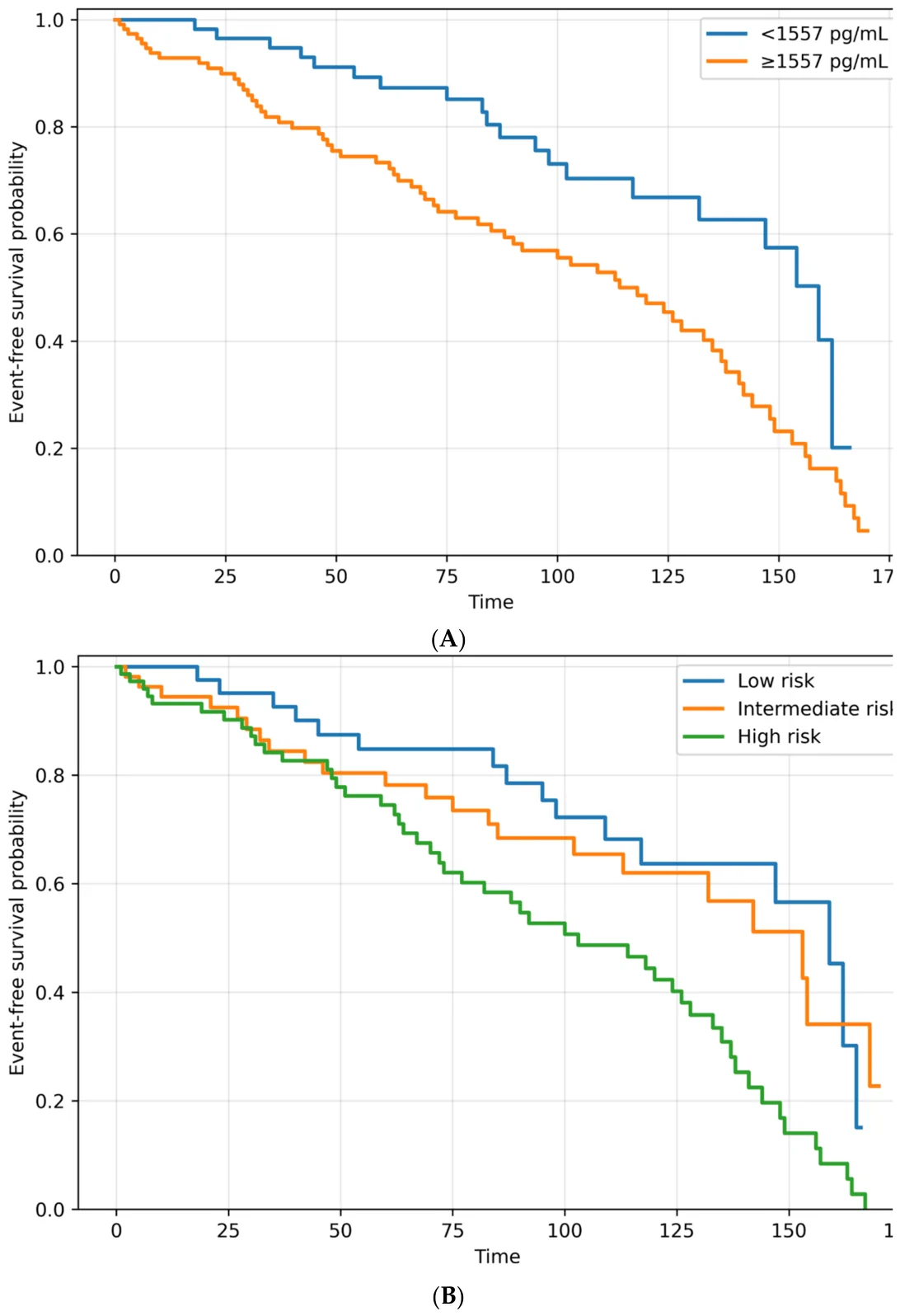
(**A**) Kaplan–Meier analysis according to 24 h hs-Troponin I concentrations. Patients with hs-Troponin I concentrations ≥1557 pg/mL had significantly lower event-free survival than those with lower concentrations (log-rank *p* = 0.0031). (**B**) Kaplan–Meier analysis according to multimarker risk categories. Event-free survival progressively decreased across increasing risk categories, with the high-risk group demonstrating the poorest prognosis during follow-up.

Among patients who experienced MACEs, the median time to first adverse event was 11 days (IQR 6.0–27.8). Kaplan–Meier analysis demonstrated significantly lower event-free survival among patients with 24 h hs-Troponin I concentrations ≥1557 pg/mL (log-rank *p* = 0.0031) (Figure 3A). A simplified four-component risk score was developed to facilitate bedside risk stratification. One point was assigned to each predictor exceeding the predefined ROC-derived cut-off value determined using the Youden index. Patients were subsequently classified into low- (0–1 points), intermediate- (2 points), and high-risk (3–4 points) categories according to the total score. The simplified four-component risk score demonstrated moderate discriminative ability for predicting 3-month MACEs, with an AUC of 0.693 (95% CI 0.615–0.768). At the optimal cut-off of ≥2 points, the score achieved a sensitivity of 73.9% and a specificity of 57.3%.

A simplified four-component score incorporating 24 h hs-Troponin I, LVEF, CRP, and TyG index was developed. MACEs occurred in 36.6% of low-risk, 39.2% of intermediate-risk, and 67.9% of high-risk patients (*p* < 0.001). The high-risk category was associated with increased odds of MACEs (OR 3.45, 95% CI 1.83–6.52, *p* < 0.001) and a higher hazard of event occurrence (HR 2.36, 95% CI 1.53–3.64, *p* < 0.001). Each one-point increase in score was associated with a 54% increase in MACE hazard (HR 1.54, 95% CI 1.25–1.91, *p* < 0.001) (Table 8, Figure 3B).

## 4. Discussion

In this prospective cohort of patients admitted to a Medical Intensive Care Unit with NSTEMI, the principal finding was that integration of markers reflecting myocardial injury, ventricular function, and inflammation improved short-term risk stratification beyond clinical variables alone. Although the individual biomarkers demonstrated only modest discriminative performance, their combined assessment identified patients at substantially different risks of adverse cardiovascular outcomes during follow-up.

Among the individual components of the model, 24 h hs-Troponin I emerged as the most informative marker. Higher 24 h hs-Troponin I concentrations were independently associated with both MACE occurrence and earlier event development, whereas admission hs-Troponin I concentrations were not associated with outcome. Lower LVEF also provided independent prognostic information, while CRP contributed additional risk information when incorporated into the multimarker model [12].

Another important observation was the high event rate during follow-up. More than half of the study population experienced MACEs within three months, and the median time to the first event was only 11 days. Cardiovascular death and hospitalization for worsening HF accounted for most adverse outcomes. Although the overall event rate appears higher than that reported in many contemporary NSTEMI cohorts, all patients in the present study required admission to a Medical Intensive Care Unit of a tertiary referral center and therefore represented a population with greater clinical complexity and higher baseline risk [13]. Furthermore, the composite MACE endpoint included hospitalization for worsening heart failure and urgent coronary revascularization in addition to cardiovascular death, recurrent myocardial infarction, and ischemic stroke, which may also have contributed to the observed event rate. Moreover, the individual components of the composite MACE endpoint differ in their clinical severity and prognostic significance; therefore, interpretation of the composite outcome requires appropriate caution. Consequently, these findings should be interpreted within the context of this selected high-risk population and may not be directly generalizable to lower-risk patients with NSTEMI. These findings emphasize the importance of early risk assessment before hospital discharge, as many adverse events occurred during the first weeks after hospitalization.

LVEF remained associated with outcome after adjustment for clinical characteristics and biomarkers [14]. This finding is consistent with the established role of ventricular systolic dysfunction as a determinant of prognosis after acute myocardial infarction. While troponin reflects the magnitude of myocardial injury, LVEF reflects its functional consequences [15]. The improvement in model performance after incorporating LVEF suggests that TTE assessment continues to provide clinically relevant prognostic information beyond biochemical markers alone [16].

Inflammation also appeared to contribute to risk stratification [17]. Although CRP did not remain an independent predictor after multivariable adjustment, its inclusion improved the model’s overall discrimination and reclassification [18]. This finding is biologically plausible given the central role of inflammation in plaque destabilization, recurrent ischemic events, and post-infarction ventricular remodeling. In contrast, ferritin and the evaluated metabolic indices provided limited additional prognostic information in this cohort. Their contribution to model performance was small and diminished after internal validation [19].

An unexpected finding was the inverse association of age and active smoking with adverse outcomes in multivariable analyses. Similar observations have occasionally been reported in populations with acute coronary syndromes and are often attributed to residual confounding or selection effects rather than to true protective associations [20]. Differences in baseline characteristics, treatment patterns, referral practices, and survivor bias may all contribute to such findings. Given the observational design and relatively limited sample size, these results should be interpreted cautiously and should not be considered as evidence of a protective effect of smoking.

The most clinically relevant finding was the improvement achieved by integrating multiple pathophysiological domains [21]. Sequential incorporation of 24 h hs-Troponin I, LVEF, and CRP improved discrimination, reclassification, and clinical utility compared with a model based solely on clinical characteristics [22]. The resulting multimarker score identified patient groups with markedly different risks of adverse outcomes while relying exclusively on routinely available clinical, laboratory, and TTE parameters [23].

Several established risk scores, particularly GRACE and TIMI, remain the cornerstone of risk assessment in patients with NSTEMI and have undergone extensive validation [24]. However, these scores do not simultaneously integrate serial assessment of myocardial injury, left ventricular systolic function, and inflammatory activity obtained during the index hospitalization [25]. Although a direct comparison with the GRACE and TIMI risk scores was not possible in the present study, the proposed multidomain model demonstrated good discriminative performance, suggesting that integrating these complementary domains may provide additional prognostic information. Whether such an approach offers incremental value beyond established risk scores should be examined in larger external cohorts [26]. Accordingly, the proposed multidomain prediction model should currently be considered complementary to established clinical risk scores, such as GRACE and TIMI, and hypothesis-generating until validated in direct comparative studies.

This study has several limitations. It was conducted at a single tertiary-care center and included a relatively modest number of patients. Follow-up was limited to three months, precluding assessment of long-term outcomes. Further studies with longer follow-up are needed to determine whether the model retains its predictive performance over longer follow-up periods. The primary endpoint was a composite of clinically distinct cardiovascular events. Established risk scores such as GRACE and TIMI could not be evaluated because several required variables were unavailable. Although the initial treatment strategy, including percutaneous coronary intervention, surgical revascularization, or conservative medical management, was recorded during the index hospitalization, changes in medical therapy and additional revascularization procedures during the three-month follow-up were not systematically collected and therefore could not be incorporated into the analyses. In addition, the study population consisted exclusively of patients admitted to a medical intensive care unit, which may limit generalizability to lower-risk NSTEMI populations. Finally, although the proposed model underwent internal bootstrap validation, the relatively modest sample size may still influence model stability. Furthermore, external validation was not available, and the findings require confirmation in larger, independent multicenter cohorts before routine clinical application.

## 5. Conclusions

In patients admitted to a medical intensive care unit with NSTEMI, 24 h hs-Troponin I was a stronger predictor of short-term adverse cardiovascular outcomes than admission measurements. Integrating 24 h hs-Troponin I, LVEF, and C-reactive protein improved risk prediction beyond clinical variables alone and enabled the development of a practical multimarker model based on routinely available parameters. These findings support the potential value of combining markers of myocardial injury, ventricular function, and inflammation for early risk stratification in high-risk patients with NSTEMI.

## Figures and Tables

**Table 1:** Baseline clinical, laboratory, metabolic, and echocardiographic characteristics of patients according to 3-month MACE status.

Variable	Total (*N* = 170)	MACEs (*n* = 88)	No MACEs (*n* = 82)	*p*-Value
Demographic and clinical characteristics				
Age, years, median (IQR)	68 (60–75)	68 (60–76)	67 (60–74)	0.780
Male sex, *n* (%)	103 (60.6)	52 (59.1)	51 (62.2)	0.680
Arterial hypertension, *n* (%)	149 (87.6)	82 (93.2)	67 (81.7)	0.041
Diabetes mellitus, *n* (%)	71 (41.8)	38 (43.2)	33 (40.2)	0.700
Active smoking, *n* (%)	85 (50.0)	37 (42.0)	48 (58.5)	0.046
Body mass index, kg/m^2^, median (IQR)	30.10 (27.46–32.05)	30.30 (27.80–32.20)	29.90 (27.30–31.90)	0.550
Markers of myocardial injury and inflammation				
hs-Troponin I at admission, pg/mL, median (IQR)	1939.5 (401.4–11,568.0)	2140.0 (476.7–12,382.3)	1521.2 (350.0–8985.3)	0.598
hs-Troponin I at 24 h, pg/mL, median (IQR)	6770.8 (990.0–18,693.8)	10,211.3 (1999.4–23,859.0)	3004.1 (624.2–14,176.6)	0.008
CRP at 24 h, mg/L, median (IQR)	23.4 (5.9–55.8)	31.8 (6.5–70.4)	12.2 (4.9–34.4)	0.022
Ferritin at 24 h, µg/L, median (IQR)	155.8 (57.7–324.3)	165.8 (68.8–334.4)	155.1 (54.3–310.8)	0.407
Metabolic marker				
TyG index, median (IQR)	9.31 (8.89–9.95)	9.39 (8.97–10.01)	9.23 (8.82–9.89)	0.248
Echocardiographic parameters				
LVEF, %, median (IQR)	49.0 (43.3–52.0)	48.5 (41.0–51.0)	50.0 (45.3–53.8)	0.032
Left atrial diameter, mm, median (IQR)	41.0 (38.0–44.0)	41.0 (38.0–44.0)	40.0 (37.0–43.0)	0.287
Interventricular septal thickness, mm, median (IQR)	12.0 (11.0–13.0)	12.0 (11.0–13.0)	12.0 (11.0–13.0)	0.551
Inferior vena cava diameter, mm, median (IQR)	18.0 (16.0–21.0)	19.0 (16.0–21.0)	18.0 (16.0–20.0)	0.416

Data are presented as median (IQR) or *n* (%). Abbreviations: CRP, C-reactive protein; hs-Troponin I, high-sensitivity cardiac troponin I; IQR, interquartile range; LVEF, left ventricular ejection fraction; MACEs, major adverse cardiovascular events; TyG, triglyceride–glucose index.

**Table 2:** Components of major adverse cardiovascular events (MACEs) during the 3-month follow-up.

Component of MACEs	*n* (%) of Total Cohort	% of All MACEs
Cardiovascular death	25 (14.7%)	28.4%
Hospitalization for worsening heart failure	29 (17.1%)	33.0%
Recurrent myocardial infarction	18 (10.6%)	20.5%
Urgent coronary revascularization	11 (6.5%)	12.5%
Ischemic stroke	5 (2.9%)	5.7%
Total MACEs	88 (51.8%)	100.0%

Abbreviations: MACEs, major adverse cardiovascular events.

**Table 3:** Prognostic performance of candidate biomarkers for prediction of 3-month major adverse cardiovascular events (MACEs).

Variable	AUC	95% CI	Optimal Cut-Off *
hs-Troponin I at admission	0.524	0.437–0.611	1885 pg/mL
hs-Troponin I at 24 h	0.618	0.532–0.697	1557 pg/mL
CRP at 24 h	0.602	0.516–0.686	30.2 mg/L
LVEF	0.595	0.513–0.679	52%
Ferritin at 24 h	0.537	0.449–0.624	164 µg/L
TyG index	0.551	0.463–0.637	9.17
TG/HDL-C ratio	0.544	0.456–0.631	1.48
Atherogenic index of plasma (AIP)	0.544	0.456–0.631	0.15

* Optimal cut-off values were determined using the Youden index. Abbreviations: AIP, atherogenic index of plasma; AUC, area under the curve; CI, confidence interval; CRP, C-reactive protein; HDL-C, high-density lipoprotein cholesterol; LVEF, left ventricular ejection fraction; TyG, triglyceride–glucose index.

**Table 4:** Independent predictors of 3-month major adverse cardiovascular events in multivariable logistic and Cox regression analyses.

Variable	OR (95% CI)	*p*-Value	HR (95% CI)	*p*-Value
Age, per SD increase	0.63 (0.43–0.92)	0.017	0.74 (0.57–0.94)	0.015
Arterial hypertension	3.32 (1.13–9.75)	0.029	2.38 (1.01–5.60)	0.046
Active smoking	0.32 (0.14–0.72)	0.006	0.49 (0.30–0.82)	0.006
hs-Troponin I at 24 h *	1.57 (1.09–2.26)	0.015	1.38 (1.07–1.78)	0.012
LVEF	0.70 (0.50–0.99)	0.046	0.88 (0.71–1.08)	0.214
CRP at 24 h *	1.36 (0.95–1.97)	0.096	1.15 (0.92–1.43)	0.223

* Log-transformed before analysis. Abbreviations: CI, confidence interval; CRP, C-reactive protein; HR, hazard ratio; LVEF, left ventricular ejection fraction; OR, odds ratio; SD, standard deviation.

**Table 5:** Multivariable logistic regression coefficients of the final multidomain prediction model for 3-month major adverse cardiovascular events.

Variable	OR (95% CI)	*p*-Value
Age, per SD increase	0.63 (0.43–0.92)	0.016
Male sex	1.15 (0.51–2.60)	0.729
Arterial hypertension	3.34 (1.13–9.80)	0.029
Diabetes mellitus	0.57 (0.28–1.14)	0.112
Active smoking	0.32 (0.14–0.72)	0.006
hs-Troponin I at 24 h *	1.56 (1.08–2.24)	0.017
LVEF	0.70 (0.50–1.00)	0.047
CRP at 24 h *	1.39 (0.96–2.01)	0.081

* Log-transformed before analysis. Abbreviations: CI, confidence interval; CRP, C-reactive protein; LVEF, left ventricular ejection fraction; OR, odds ratio; SD, standard deviation.

**Table 6:** Performance of sequential multidomain prediction models for the prediction of 3-month major adverse cardiovascular events.

Variables Included	AUC (95% CI)
Clinical variables †	0.665 (0.582–0.739)
Clinical variables + hs-Troponin I at 24 h	0.730 (0.654–0.801)
Clinical variables + hs-Troponin I at 24 h + LVEF	0.745 (0.670–0.814)
Clinical variables + hs-Troponin I at 24 h + LVEF + CRP (final model)	0.759 (0.685–0.826)
Final model + ferritin + TyG index	0.762 (0.689–0.829)

† Clinical variables included age, sex, arterial hypertension, diabetes mellitus, and active smoking. Abbreviations: AUC, area under the curve; CI, confidence interval; CRP, C-reactive protein; LVEF, left ventricular ejection fraction; TyG, triglyceride–glucose index.

**Table 7:** Internal validation, calibration, and reclassification performance of the final multidomain prediction model for the prediction of 3-month major adverse cardiovascular events (MACEs).

Metric	Clinical Model	Final Multidomain Model
Apparent AUC	0.665	0.759
Optimism-corrected AUC	0.623	0.717
Optimism	0.042	0.042
Brier score	0.229	0.201
Hosmer–Lemeshow *p*-value	0.280	0.410
Calibration slope	1.000	0.930
Calibration intercept	0.000	−0.040
IDI	0.000	0.106
Continuous NRI	0.000	0.586

Bootstrap internal validation was performed using 1000 resamples. Abbreviations: AUC, area under the curve; IDI, integrated discrimination improvement; NRI, net reclassification improvement.

**Table 8:** Multimarker risk score and risk of 3-month major adverse cardiovascular events.

Risk Category	Score	Patients (*n*)	MACEs, *n* (%)	OR (95% CI)	HR (95% CI)
Low risk (reference)	0–1	41	15 (36.6)	Reference	Reference
Intermediate risk	2	51	20 (39.2)	1.12 (0.51–2.47)	1.08 (0.57–2.05)
High risk	3–4	78	53 (67.9)	3.45 (1.83–6.52)	2.36 (1.53–3.64)

Abbreviations: HR, hazard ratio; MACEs, major adverse cardiovascular events; OR, odds ratio.

## Data Availability

The data presented in this study are available on request from the corresponding author. The data are not publicly available due to privacy and ethical restrictions.

## Abbreviations

The following abbreviations are used in this manuscript:

AIP	Atherogenic Index of Plasma
AUC	Area Under the Receiver Operating Characteristic Curve
BMI	Body Mass Index
CI	Confidence Interval
CRP	C-reactive Protein
DM	Diabetes Mellitus
ECG	Electrocardiography
HF	Heart Failure
HR	Hazard Ratio
hs-Troponin I	High-Sensitivity Cardiac Troponin I
HTN	Arterial Hypertension
IDI	Integrated Discrimination Improvement
IQR	Interquartile Range
LVEF	Left Ventricular Ejection Fraction
MACE	Major Adverse Cardiovascular Events
NRI	Net Reclassification Improvement
NSTEMI	Non-ST-Segment Elevation Myocardial Infarction
OR	Odds Ratio
ROC	Receiver Operating Characteristic
TG/HDL-C	Triglyceride-to-High-Density Lipoprotein Cholesterol Ratio
TTE	Transthoracic Echocardiography
TyG	Triglyceride–Glucose Index
